# Evaluating a Topical Adjunctive Post Submental ATX-101 (Deoxycholic Acid) Injection for Improved Recovery: A Single-Center, Double-Blind, Randomized Controlled Pilot Study

**DOI:** 10.1093/asjof/ojab028

**Published:** 2021-06-29

**Authors:** Sachin M Shridharani

**Affiliations:** Division of Plastic and Reconstructive Surgery, Washington University School of Medicine, St. Louis, MO, USA

## Abstract

**Background:**

Optimizing postprocedural recovery and outcomes for patients is the aim for all physicians. TransFORM Body Treatment with TriHex Technology (TFB) is a topical product that aids in the elimination of fat particles created during procedures and the reduction of associated inflammation, thus speeding up postprocedure recovery time.

**Objectives:**

Evaluation of postprocedural symptoms, signs, and healing following submental deoxycholic acid (DCA) injections in combination with TFB.

**Methods:**

Participants received 2 treatments of submental DCA injections. Posttreatment 1, every participant received TFB to apply twice daily to the submental area. Follow-up visits included weeks 1, 2, and 4. After week 4, participants discontinued TFB for 30 days before the second treatment. At the second treatment visit, participants were randomized to receive either TFB or a bland moisturizer to apply twice daily with the same follow-up visits as posttreatment 1. Induration measurements, submental fullness grading, and standardized photography were captured at every visit. At all follow-up visits and before treatment 2, investigator assessments and participant assessments were completed.

**Results:**

Posttreatment 2, investigator assessments of edema and induration decreased in participants using TFB at weeks 1 and 2 compared with the bland moisturizer. Induration measurements objectively showed a statistically significant reduction at week 2 (posttreatment 2) in participants using TFB compared with the bland moisturizer. Furthermore, participants reported less tenderness and soreness in the TFB group over the bland moisturizer.

**Conclusions:**

Investigator assessments, participant query, and objective induration analyses have demonstrated that the use of TFB post DCA injections may reduce induration, edema, and discomfort associated with this procedure.

**Level of Evidence: 2:**

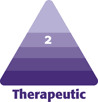

The ideal aesthetic procedure would be hallmarked by several facets: longevity of the results, least invasive option, and minimal downtime. Clinicians recognize that this holy grail is not often possible; however, measures to improve the patient experience by decreasing the duration of adverse events and/or downtime lead to increased treatment adoption and patient compliance. Deoxycholic acid (DCA) (ATX-101; KYBELLA, Allergan, Inc., Dublin, Ireland) is used to reduce and improve the appearance of submental fat and the jowls.^1,2^ However, common adverse reactions from the use of DCA include edema, induration, pain/discomfort, and erythema, which may last up to a month or longer. Post injection, recovery time is a concern for patients as well as physicians who would prefer to see a decrease in injection site recovery time especially in patients requiring repetitive treatment sessions.

DCA disrupts adipocyte membranes leading to irreversible cell breakdown (adipocytolysis).^2-7^ After adipocytolysis, an inflammatory response is induced, with macrophage recruitment and phagocytosis, through which cellular debris is cleared over time.^2-7^ These lipid droplets are thought to be inflammatory in nature contributing to the skin induration seen post injection.^8^ In addition, the destructive inflammatory nature of DCA itself also results in a significant inflammatory response. TransFORM Body Treatment with TriHex Technology (TFB) (Alastin Skincare, Inc., Carlsbad, CA, USA) improves lipid droplet dissolution, facilitated through a liposome delivery system, and the actives are deposited into the base of the hair follicle where this reservoir continually delivers the product to the dermal white then subcutaneous white adipose tissue. Hexapeptide-11 has been proven to accelerate (upregulate) the process of autophagy, encouraging lipid droplet breakdown. In vitro modeling shows upregulated macrophage recruitment to damaged fat cells with in vivo trials confirming increased and hastened fat volume reduction.^8-10^ In a recent study, patients using TFB had a noticeable reduction in postprocedural soft tissue changes and improved patient-reported recovery outcomes compared with patients not using the topical.^11^ TFB has also been clinically shown to increase comprehensive collagen and elastin stimulation, as well as enhance overall hydration and barrier function.^8^ This study evaluated recovery post submental DCA injections in combination with the use of TFB compared with a bland moisturizer.

## METHODS

This single-center, double-blind, randomized controlled pilot trial evaluated the use of TFB post submental DCA injections. This study was conducted from September 2019 to January 2020. Ethics board approval was not required. Male and female patients, with clearly visible submental subcutaneous fat and with soft, pliable tissue of sufficient volume for treatment, were included. Those with previous fat reduction procedures or implants in or near the treatment area, scars, excessive laxity, previous surgery in the treatment region, and/or any contraindications to DCA usage, as determined by the physician, were excluded from participating in the study. Women pregnant, lactating, or planning on becoming pregnant during the study duration were also excluded. Participants were consented before any study procedures, and the study was conducted under applicable regulations in accordance with Good Clinical Practice. 

At visit 1, preprocedure, standardized photography was completed after SkinFibroMeter (induration) measurements were performed, and submental fullness was graded. Participants received submental DCA injections with an average injected volume of 8 mL. Postprocedure, all participants were given TFB in a blinded bottle to use twice daily to the treatment area. Participants returned at weeks 1, 2, and 4 post treatment for follow-up and assessments. At each follow-up visit, the following was performed: standardized photography, SkinFibroMeter measurements, investigator assessments, participant assessment, and recording of adverse events and concomitant medications. At the week 4 follow-up visit, participants returned the TFB and discontinued use for 30 days before treatment 2. At treatment 2, preprocedure photography and assessments were completed, and then DCA injections performed. Participants were then randomized to receive either TFB or Cetaphil Lotion—bland moisturizer (Galderma, Fort Worth, TX, USA), in an identical blinded bottle, to apply twice daily, and return to the office for follow-up visits at weeks 1, 2, and 4. Follow-up procedures posttreatment 2 were the same as posttreatment 1. The average volume injected at treatment 2 was 8.8 mL for the TFB group and 6.4 mL for the bland moisturizer group. Treatment 2 demographics are included in Table 1.

**Table 1.** Treatment 2 Demographics

TFB							Total
Gender/age (yr)	Female age: 36	Female age: 63	Male age: 36	Male age: 36	Female age: 29	Female age: 47	4 F, 2 M Mean age: 41
Injected mL	8.8	8.8	11	13.2	4.4	6.6	Mean: 8.8
Bland moisturizer							Total
Gender/age (yr)	Female age: 25	Female age: 30	Male age: 40	Female age: 61	Female age: 40	Participant d/c	4 F, 1 M Mean age: 39
Injected mL	6.6	4.4	7.8	6.6	6.6		Mean: 6.4

d/c, discontinued; F, female; M, male; TFB, TransFORM Body Treatment.

### Investigator Assessments

At weeks 1, 2, and 4 post both DCA treatments and before the second DCA treatment, the investigator assessed the submental treatment area for induration, edema, erythema, bruising, and pain. Each assessment was graded using a (0-4) point scale. The visual analog scale (VAS), a (0-10) point scale, was used to score pain. Submental fullness was graded at every visit using the clinician-reported submental fat rating scale (CR-SFRS),^7,12^ a (0-4) point scale. At the second treatment, the investigator was blinded as to which topical the participants were applying. Scales utilized are available in the Appendix (available online at www.asjopenforum.com).

### SkinFibroMeter Measurements

The SkinFibroMeter (Delfin Technologies USA, Miami, FL, USA) is an instrument to assess tissue stiffness in quantitative units.^13,14^ It has been validated against measurements with industrial standards and in clinical studies against palpation and histological findings. Superficial induration manifesting in skin and upper subcutis can be quantified with an indentation principle, noninvasively, without alterations in measured tissue structure. Measurements were taken at every visit, in triplicate, at 3 locations (right lateral [RL], midline [MID], and left lateral [LL]) over the submental treatment area to measure induration.

### Statistical Analysis

Treatment 1 (TFB): Analysis was performed using 1-sample *t* tests to detect significant changes in the outcomes from baseline to week 4.

Treatment 2 (TFB or Bland Moisturizer): Analysis was performed using 2-sample *t* tests to compare changes in the outcomes from the treatment 2 visit to week 4, posttreatment between TFB vs bland moisturizer recipients in order to detect the effects of the topical product on edema and induration.

## RESULTS

Twelve participants, 3 males and 9 females, ages 25-63 (mean age 42), Fitzpatrick Skin Types II-V, enrolled in this study. One participant discontinued before the second treatment due to a family death.

### Treatment 1 (TFB)

Since all participants were given TFB post the first treatment of DCA, a 1-sample *t* test was used to detect significant changes in the outcomes from baseline to week 4. The mean score of CR-SFRS, a measure of submental fullness, was decreased by −0.8 units from baseline to week 4. This decrease was statistically significant (*P* = 0.0020). These results were anticipated and served as baseline comparators for treatment 2.

### Treatment 2 (TFB or Bland Moisturizer)

All participants were randomized to receive either TFB or bland moisturizer posttreatment 2. Therefore, a 2-sample *t* test was performed to compare the changes from treatment 2 to 4 weeks post between recipients of TFB or bland moisturizer to detect the postprocedural recovery effects.

### Investigator Assessments

**Figure 1.**
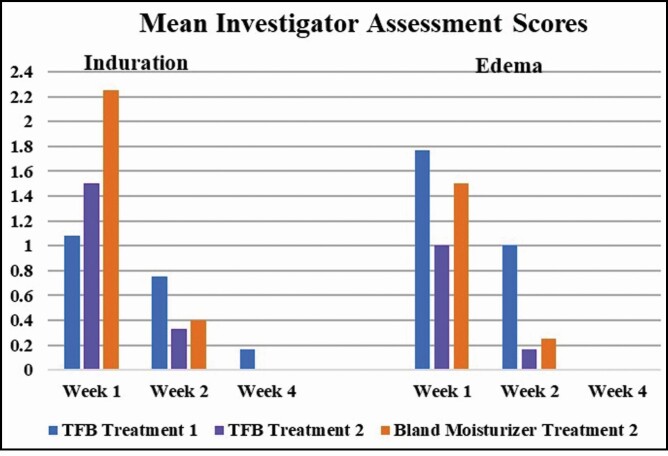
Mean investigator assessment for induration and edema posttreatments 1 and 2. TFB, TransFORM Body Treatment.

After treatment 2, less edema and induration were noted by the blinded investigator assessment in participants using TFB (Figure 1). In fact, at 1-week posttreatment 2, the degree of edema was equivalent to that of 2 weeks posttreatment 1.

**Figure 2.**
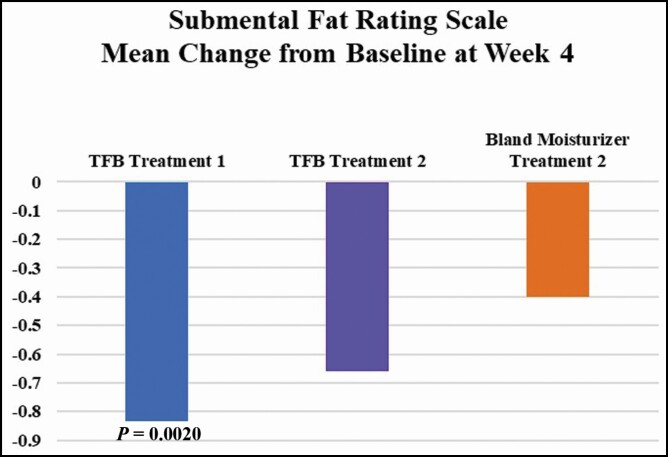
Mean change from before each treatment of the investigator assessment of submental fullness (CR-SFRS). A decrease in the scale indicates an improvement. CR-SFRS, clinician-reported submental fat rating scale. TFB, TransFORM Body Treatment.

At week 1, posttreatment 2, participants using TFB had 44% less edema than posttreatment 1 compared with 15% less edema in the participants using the bland moisturizer (Figure 1). Investigator assessment of induration followed a similar pattern. However, due to the brief interval between treatments 1 and 2, the investigator noted all participants to have residual induration likely skewing the new baseline for treatment 2 and leading to a higher induration score at week 1 posttreatment 2. At 4 weeks posttreatment 1, participants had a statistically significant decreased score on the CR-SFRS, which trended through posttreatment 2 for participants using TFB (Figure 2).

Erythema, bruising, and pain were not observed posttreatment 2 in either group, and in posttreatment 1, there was 1 incidence of pain and 2 incidents of mild bruising.

### SkinFibroMeter Measurements

**Figure 3.**
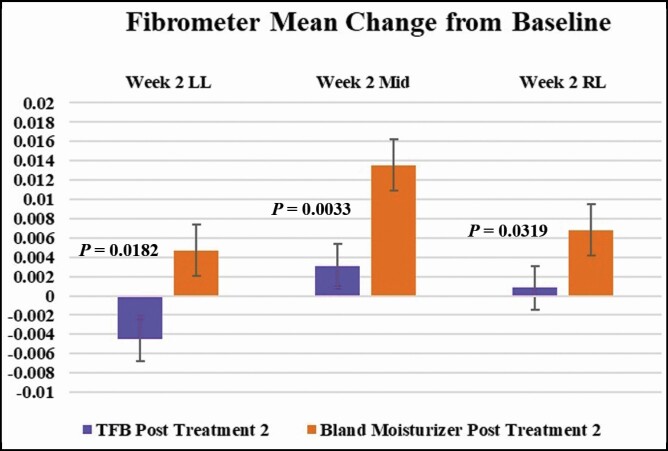
An increased score indicates increased induration from baseline. A decreased score indicates a return to baseline or lower than the induration measurement before the treatment. All measurements were taken in triplicate in the following areas: LL, submental left lateral; MID, submental midline; RL, submental right lateral; TFB, TransFORM Body Treatment.

Posttreatment 2, participants using TFB had statistically significant less induration than participants using bland moisturizer in all 3 areas measured submental at week 2 (Figure 3):

- The mean scores RL at week 2 after treatment 2 were 0.00033 among the 6 TFB recipients and 0.0068 among the 5 bland moisturizer recipients. This difference was statistically significant (*P* = 0.0319).
- The mean scores MID at week 2 after treatment 2 were 0.0031 among the 6 TFB recipients and 0.014 among the 5 bland moisturizer recipients. This difference was statistically significant (*P* = 0.0033).
- The mean scores LL at week 2 after treatment 2 were −0.0045 among the 6 TFB recipients and 0.0047 among the 5 bland moisturizer recipients. This difference was statistically significant (*P* = 0.0182).

**Figure 4.**
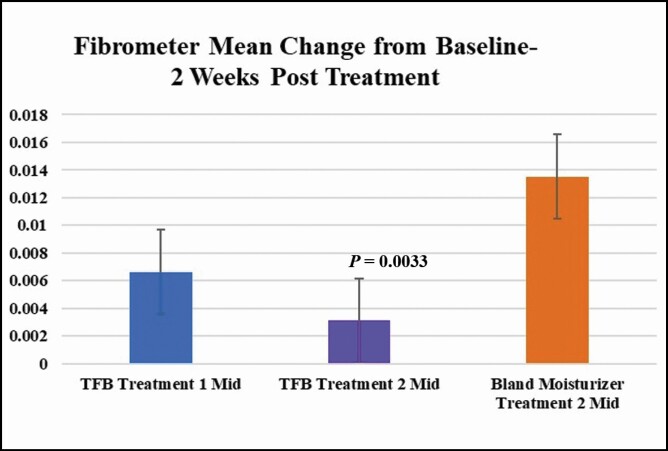
Induration measurements 2 weeks posttreatment MID (submental midline) (TFB first treatment average 0.006625, vs TFB second treatment 0.0031, vs bland moisturizer 0.013524). TFB, TransFORM Body Treatment.

Lastly, if we take the midline score averages (the area most affected), it is very apparent that 2 weeks post the second treatment, which should have less induration than the first, does so with the TFB group (TFB first treatment average 0.006625 vs 0.0031). However, when comparing this score with the bland moisturizer at the same time point, we observe a statistically significant difference (TFB first treatment average 0.006625 vs TFB second treatment 0.0031 vs bland moisturizer 0.01352) (Figure 4).

**Figure 5.**
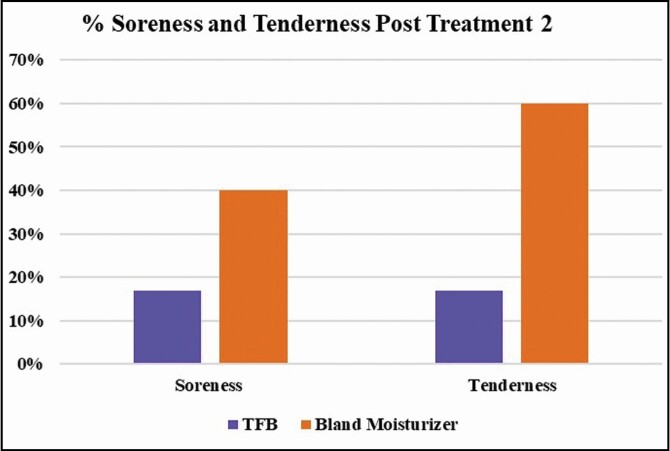
Participant-reported adverse reactions posttreatment 2. TFB, TransFORM Body Treatment.

Of the participant-reported adverse reactions, posttreatment 2, the greatest difference was tenderness and soreness with 17% reported in the TFB group compared with the bland moisturizer group with 60% tenderness and 40% soreness (Figure 5). All participants experienced numbness posttreatments 1 and 2, excluding 1 participant posttreatment 2, randomized to TFB. There was no marginal mandibular paresis or alopecia reported.

## DISCUSSION

DCA is a popular procedure for the dissolution of submental fat, and it is of importance for physicians and patients to curtail the downtime posttreatment, thereby improving the patient experience.

**Figure 6.**
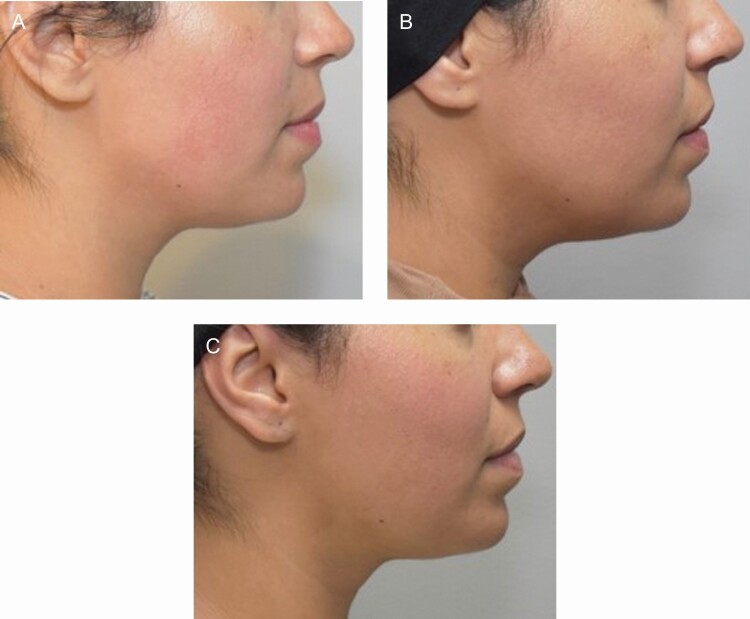
Participant 3, 36-year-old female. Applied TFB post both treatments. (A) Baseline, (B) 2 weeks posttreatment 1, and (C) 2 weeks posttreatment 2 showing less induration and swelling at treatment 2 than at treatment 1. Treatment 1, 8.8 mL injected, and Treatment 2, 8.8 mL injected. TFB, TransFORM Body Treatment.

**Figure 7.**
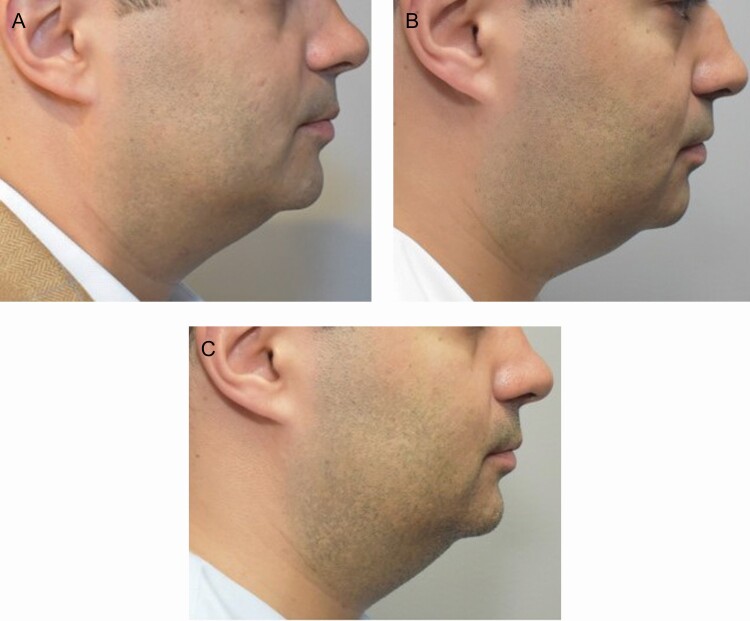
Participant 4, 40-year-old male. Applied TFB posttreatment 1 and bland moisturizer posttreatment 2. (A) Baseline, (B) 2 weeks posttreatment 1, and (C) 2 weeks posttreatment 2 showing more induration and swelling at treatment 2 applying bland moisturizer than at treatment 1 with TFB. Treatment 1, 8.8 mL injected, and treatment 2, 7.8 mL injected. TFB, TransFORM Body Treatment.

DCA disrupts the membranes of adipocytes through solubilization of the membrane lipids, leading to cell breakdown, which induces a local inflammatory response that clears the adipocyte debris.^1-7^ The peptides in TFB stimulate the autophagic processes and further breakdown the lipid droplets and encourage macrophage clustering and phagocytosis, thereby speeding up the recovery post procedure.^8-11^ The decreased induration and edema in participants using TFB at weeks 1 and 2 posttreatment 2 demonstrate the effects of applying TFB post procedure. Although patients often have a better reaction on repeat injections, it is noteworthy that a topical preparation can further reduce the anticipated adverse events. The comparison of the 2 products, using objective SkinFibroMeter assessments after treatment 2, further validates the blinded investigator assessments of lessened edema and induration in all areas. The SkinFibroMeter consists of a 1.25-mm length indenter and 2 force sensors. The device is briefly pressed against the skin and the contact force is registered. The indenter imposes a constant deformation when the instrument is in full contact with the skin. The skin and the underlying upper subcutis resist the deformation, and the induration value in Newtons (N) is determined. SkinFibroMeter measurements showed a statistically significant reduction in induration at week 2 (posttreatment 2) in participants using TFB compared to the bland moisturizer. Furthermore, participants using TFB reported less tenderness and soreness over the bland moisturizer group. As anticipated, the greatest change in submental fullness was apparent 4 weeks following the first treatment. The second treatment would be expected to have less dramatic fullness changes, but, once again, it is noteworthy that the TFB-treated participants showed a better outcome at the 4-week time point posttreatment 2. Figure 6 is representative of a participant applying TFB post both treatments and Figure 7 represents a participant applying TFB post treatment one and bland moisturizer post treatment 2.

TFB has also been shown post liposuction to hasten the inflammatory phase and initiate anti-inflammatory genes in the early recovery period and to stimulate extracellular matrix (ECM) remodeling and wound healing in the longer 4-week postsurgical period.^15, 16^ This corresponds well with the observations in this study. Hastened decreased inflammation is likely manifesting as less edema and induration, and the ECM remodeling with lipid droplet dissolution may improve DCA efficacy and aesthetic outcomes.

This pilot study was designed to create a baseline view of postprocedural application with TFB, using treatment 1 as an internal control since historically there are more reported adverse events after treatment 1 and variability in patient severity. After a washout period, participants were randomized to enable experiential comparisons between the use of the 2 products. It was undertaken to assess the impact on patient experience and postprocedural recovery related to submental injections of the FDA-approved location of DCA, which also happens to be the most frequently injected area. It was not intended to assess the efficacy of DCA or long-term outcomes. The limitations of this study include sidedness, which is not possible in the neck. Future studies could be designed to treat isolated pockets of adipose deposition and use the same patient’s anatomy as an internal control. Additionally, a more robust participant size, randomization posttreatment 1, and an increased time period of 60 days in between treatments to ensure that all adverse events have resolved. The goal of this study, however, was to assess the efficacy of TFB vs a bland moisturizer posttreatment 2 after all participants had used posttreatment 1, so as not to discern that some participants had more postprocedural adverse reactions than others.

## CONCLUSIONS

Investigator assessments and objective induration analyses have demonstrated that the use of TFB post DCA injections may reduce induration, edema, and discomfort associated with this procedure. Improvement in patient experience is a welcome adjunct to any procedure, particularly those needing repetitive sessions.

## Supplementary Material

ojab028_suppl_Supplementary_Appendix
