# Supplementary material for: Evaluating a Topical Adjunctive Post Submental ATX-101 (Deoxycholic Acid) Injection for Improved Recovery: A Single-Center, Double-Blind, Randomized Controlled Pilot Study
Source: Aesthet Surg J Open Forum. 2021 Jun 29;3(3):ojab028. doi: 10.1093/asjof/ojab028 (PMC8353883; doi:10.1093/asjof/ojab028)

**Scales for Grading**

**Induration**

**Grades 0-4**

0 is none

1 is barely perceptible induration

2 is slight but definite induration

3 is moderate-clearly perceptible induration

4 is severe- marked induration

**Edema**

**Grades 0-4**

0 is none

1 is barely perceptible edema

2 is slight edema-edges of area well defined by definite raising

3 is moderate edema-edges of area well defined by clearly perceptible raising

4 is severe edema extending beyond the area treated

**Erythema**

**Grades 0-4**

0 is none

1 is barely perceptible erythema

2 is slight but definite erythema (pink)

3 is moderate-clearly perceptible erythema (dull red)

4 is severe- marked erythema (deep or bright red)

**Bruising**

**(color)**

4Red

3Dark blue, Purple or Black

2Yellow or Green

1Yellowish-Brown or Light Brown

**VAS- Pain/Discomfort**


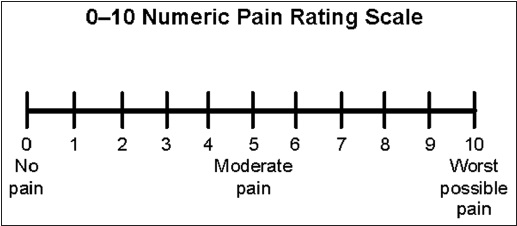


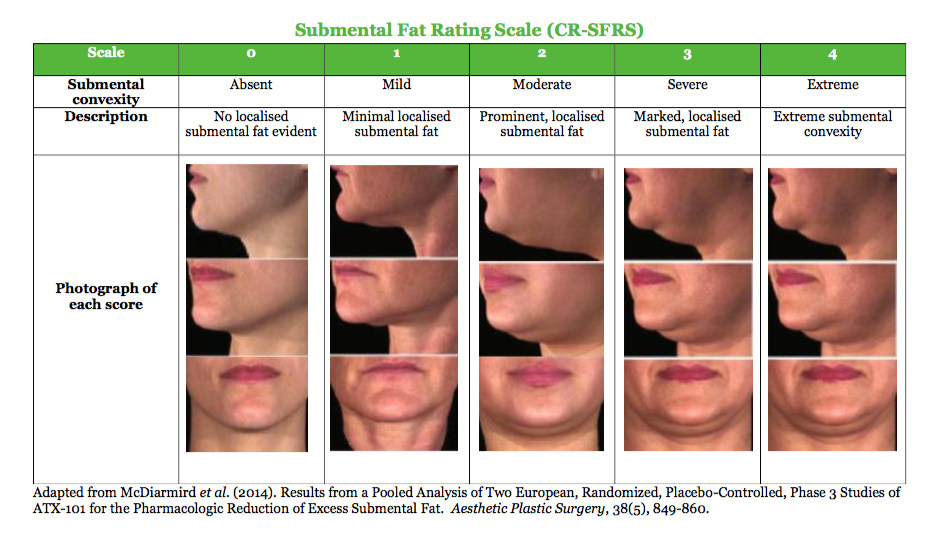

Supplement: ojab028_suppl_Supplementary_Appendix [file ojab028_suppl_supplementary_appendix.docx]
